# Origami based ultraviolet C device for low cost portable disinfection- using a parametric approach to design

**DOI:** 10.1557/adv.2020.425

**Published:** 2020-12-01

**Authors:** Samriddho Ghosh, Mainak Ghosh

**Affiliations:** 17 Undergraduate student, Department of Architecture, Jadavpur University, Kolkata, India; 27 Department of Architecture, Jadavpur University, Kolkata, India

## Abstract

Microorganisms can be present in common equipment of practice, which spread Healthcare-associated infections (HALs), generating major health issues, not only in the hospital environment but outside as well. Especially in this impending crisis due to SARS CoV-19 virus, disinfection (in general) turns out to be of paramount importance and portable disinfection in particular. The rampant spread of the COVID 19 virus, has pushed thinkers to come up with unique disinfection solutions that can help curb the spread of the virus in other places other than the medical facilities. The article aims to establish a design of a portable ultraviolet c disinfectant device that is based on the core principles of origami- the ancient Japanese art of paper folding. Resorting to origami has helped to make the device ultra-portable, robust and can be easily manufactured in order to be commercially produced as an inexpensive alternative to the cost portable disinfection devices in the market.

## INTRODUCTION

Healthcare associative Infections (HALs), these are responsible for prolonging hospital stay, cause long-term disability and additional costs for health systems, and preventable deaths. The possibility of multidrug resistance increases if HALs aren't arrested in due course. Such pathogens like the *SARS CoV-19* are transmitted by hand contact or patient to patient contact that leads to the proliferation of the same. These virus can be found inside the body, on the skin, on daily objects. Owing to this fact, surface disinfection has found its way as a mandatory procedural activity in not only hospitals but also in the personal care sector. This demand of disinfection has ameliorated, in 2020 particularly owing to the COVID 19 crisis. Presently such devices have not taken a sector oriented approach, whereby the root of the spread has not been detected. Targeting those segments, with lack of awareness or lack of facilities shall be a prudent way to curb the pandemic [11]. As spoken earlier, this device is primarily made for those who belong to this sector. As the design helps, it can be a solution to the immediate spread on a very grass root level.

Two widely used types of disinfection that are pervasive are the alcohol-based disinfectant and ultraviolet (UVC) disinfection, to be precise ultraviolet sterilisation. Various UVC disinfection products are available commercially. Design innovations pertaining to such products are limited to portability and structure. One very important limitation is the cost at which it is sold. This typically varies due to size, from $50 to $180, in general. Actual prices may vary. Due to such a price range, it becomes impossible for the economically backward sector to have access to such sophisticated instruments for personal care and hygiene outside the hospital environment. The design presented aimed to cut down on costs and make the device more portable with respect to extreme conditions.

To understand the setting better, let us consider Madagascar- a country where people live on an average of $1.90 per day (2), it becomes increasingly difficult day by day in order to provide for basic sanitation and disinfection facilities, on a personal scale until institutional help is provided. Due to the lack of a renewable energy policy in Madagascar (1), basic necessities such as electricity, turns out to be impedance on the path to growth. With such obstacles, it becomes impossible for such locations to utilise sophisticated technology in order to cater to their safety and needs. Hence, the device designed in this article considers all such obstacles, to come up with a best fit product to curb such hindrance, in the personal healthcare sector.

## RESEARCH METHODOLOGY

While analysing the problem, the disinfection device aims to solve, a background understanding of the situation on ground is of paramount importance. In times of the Corona virus pandemic, there exists multiple underdeveloped and developing nations, where basic disinfection and sanitation facilities cannot reach to its extreme parts, majorly due to lack of resources, in the form of economic strife, logistical inaccessibility and lack of literacy. To overcome these encumbrances and to come up with a device that shall not only disinfect daily objects, from a personal point of use but also aim to bypass these impedances, to deliver basic disinfection facilities, in the aforementioned places.

The process entails a parametric analysis based approach, suiting the various aspects of the product- size requirement, compressibility, etc. At first the task at hand is understanding as to which origami mechanism shall be employed in this device- origami mechanisms are a type of compliant mechanisms which follow the kinematics in origami models.

This device specifically proposes a requirement of an origami bellow- these are a type of complaint mechanisms- in order to achieve required displacement, force or motion, they employ deflection of flexible members, hence are compliant. Compliant mechanism are able to reduce complex, multi segmented mechanisms into a single part by resorting on deflection for motion instead of rigid links and joints [3]. Lesser parts, ease of replication, reduction in cost, assembly, friction, wear and tear, high precision are some of the advantages of such a mechanism now. These reasons enable deployment of origami structures in harsh conditions be it that of space or in interior Africa. While designing the device, a set of parameters shall be laid out circumlocuting around which the design is developed. These parameters are mainly structural and proportion dependant ones, enabling the structural fulfilment of the product with regard to both functionality and ergonomics.

In modern times, computer aided processes involving software like Grasshopper (Grasshopper is a visual programing language developed by Robert McNeel and Associates)is used for parametric modelling, however we use a more manual iterative process with limited options, for clarity of the parameter dependent process and how each one is affecting the entire product. Design options can be generated manually via altering parametric values from the start to end of the design process. Manually arduous processes of generating curvilinear and non-Euclidean forms can be shown, however in such a case, it's likely we shall not encounter it. Although, the entire process of parametric design is approached in a computational methodology, the conceptual design process is being carried out manually, for the said reasons above [4].

## THE DESIGN

Origami based mechanisms could prove to be a viable solution to the need for developing a lightweight, highly-compressible deployable cylinder to house objects, materials and to be portable and transportable at the same instant. The origami cylinder, commonly referred as bellows, is a collapsible device which can expand to be deployed and contract when not in use for ultra-portable storage solutions [5]. The ideal material for the body of the disinfection device is synthetic tear resistant paper, owing to its structural features and its light weight. Since origami is an art revolving around paper, and since paper acts as a very good option in such a product, it is prudent to proceed with such. Cost is of paramount importance in this regard, and since manufacturing can be fast and relatively simple than metal or any other form of polymer, hence paper is encouraged.

However, with the massive benefits of papers, comes its challenges too. Primarily, the quality is of grave concern, the preferred grade of tear resistant paper, say consists of imperfections, can accelerate fatigue in the bellow, that can result in reduced user experience time, hence serve as a failed product. Secondly, water proofing is an issue with the cost kept on mind. Using a water resistant coat on the outer surface of the device, shall contribute to increased manufacturing cost. But if used in extreme conditions, this requirement is paramount for a complete user experience. Next issue that can arise is easy deformation into something unwanted due to the structural nature of paper. Rough handling of the device can result into something like this. Precautions to prevent such incidents must be undertaken. Decreasing the likelihood of failure is another important aspect of the design. The bellows is less likely to fail at any single vertex, by minimizing the number of vertices. [3].

There are various patterns with respect to which one could be used to make the origami bellows, starting off with the Kresling fold pattern and the Accordion fold pattern [3]. Both of them are not rigid foldable structures and they have a clear cross sectional area within the annulus. However, due to fatigue in deflection cycling after a low number of repeated stress applications, the Accordion fold pattern is not a suitable pattern to use in this case [6].

One tessellation of the Kresling pattern is shown in figure 1, where the dependent parameters are a, b, c, Δ. Equations explaining the Kresling tessellation unit are given below [3];

$$a = D \sin(\pi/n) \quad \ldots\ldots\ldots\ldots\ldots\ldots\ldots\ldots\ldots\ldots \text{(1)}$$

$$b = D \sin(\arccos(d/D) - \pi/n) \ldots\ldots\ldots\ldots\ldots \text{(2)}$$

$$c = D \sin(\arccos(b/D) + \pi/n) \ldots\ldots\ldots\ldots\ldots \text{(3)}$$

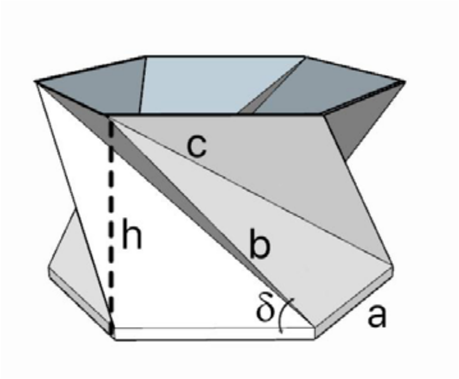

Figure 1- One storey of the Kresling fold pattern n being an independent parameter, has greater implications on the design. It is defined as the number of panels present in one storey where the number of storey for the bellow is denoted by s. In such a case the number of panels and the mechanical strain in the bellow follow a relation- As the number of panels in each storey increases, the strain amount decreases. An ideal value of n is chosen; n=9 - which ensures, significantly lesser strain than lower n values [6]. After we understand the parameter that entail this design, now the focus shifts on to the parametric permutation, as to how to bring about the ideal bellow for the point of use this article focuses on.

Another, concern when we are referring to the portability aspect of the device, is the compressibility of the bellow- a primary reason why such an origami pattern was selected. Depending on the material and requirement of deployment, when in use- the bellow can be designed accordingly altering the height of each storey (h) and the deployment angle (δ).[3]

$$h = b \sin\delta \ldots\ldots\ldots\ldots\ldots\ldots\ldots\ldots\ldots\ldots\ldots\ldots \text{(4)}$$

If there is a reduction in storeys required, keeping intact the overall linear dimension of the device, then we have to increase the height of each storey, consequently increasing the deployment angle. The height of storey and the deployment angle are independent of any other functional parameter, whereas they are interdependent parameters, when considered together. While different materials of the bellow have different compressibility, the one's made out of metals have a compressibility of approximately 66%, but in this scenario we use, paper where the compressibility percentage varies from 90-92 % approximately [12]. For bellows the compressibility is computed as the ratio of the change of height after being deployed to the deployed height of the bellow;

Compressibility = t-t'/t ……………….. (5)

Where, t = Deployed height t'= Compressed height.

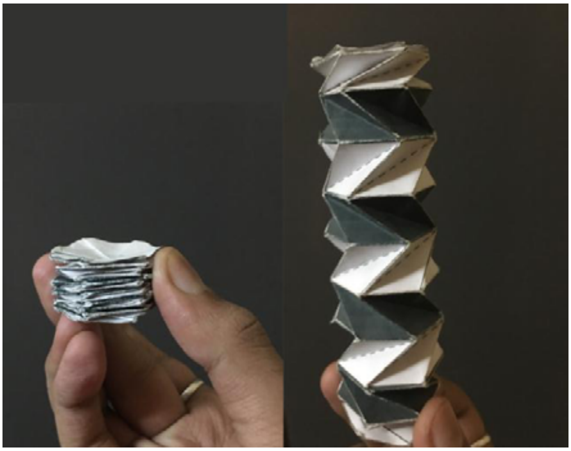

Figure 2- Kresling patterned bellow in contracted and expanded position.

Here, decreasing the likelihood of failure of the bellow is a major aspect of the design, since it's made out of paper, can be ensured if the bellow have significantly reduced number of vertices [13]. As discussed earlier, this can be achieved by reducing the number of layer storey and increasing the deployment angle, enforcing lesser buckling points for fracture and helping through the stress distribution, during deflection cycling. These structural features, primarily depend on the material used, which in turn is pivoted around the selection of the design, driven by cognitive and intuitive knowledge [10]. Based on the application and demands posed by the circumstances, materials can be appropriately altered to fit the need.

## CONSTRUCTION

The rationale behind using such complex paper geometry to achieve pragmatic advantages over traditions disinfection cum storage devices, is to be able to inject the product into locations, where a sophisticated metal or HDPE disinfection device is a farfetched possibility, both economically and logistically. Hence storage of daily objects, like keys, chains, pens and other necessary items of work can find their safe house, into the origami bellow, equipped with UV lamps to kill pathogens present on the surface of the objects the bellow houses. Far UVC lamps of wavelengths 207-222 nm should be enough to destroy the SARS-CoV-2, [7] also since the entire disinfection process takes place in the closed paper bellow, which essentially acts as a closed chamber, the UVC cannot be charged with deleterious effects on humans, since precautions are to be taken for grid locking minimum light leaking possibilities [14].

2 plastic parts that shall house 3 Far UVC LEDs on each end shall be 3D printed, out of fine PLA, and to be attached at the either end. Essentially it forms a collapsible storage space, that as the ability to disinfect, without remote contact of any human. At the rear end, the battery is housed, which is essentially a non-rechargeable 1.2 V cell, used to power the LEDs and can't disposed, once it runs out. Since the objects to disinfect are to be inserted into the interior of the bellow from A, hence the battery and the switch are to be housed on the rear side, i.e. B.

With two pieces of plastic and a collapsible paper structure, this forms a collapsible system of housing objects- the embodiment when in the collapsed version, is functionally inactive, and however is extremely portable in terms of scale. By proportional alteration of the said parameters, in the origami pattern the bellow can be scaled onto any volume, depending on the requirement of the customer, organisation or even demography keeping in mind the COVID-19 scenario. Non-tearable paper, being a robust material for the extreme conditions it can function in, and the portability enabler is an important reason for driving down the overall cost of the product, making it accessible to the common masses in target countries as discussed earlier in the article.

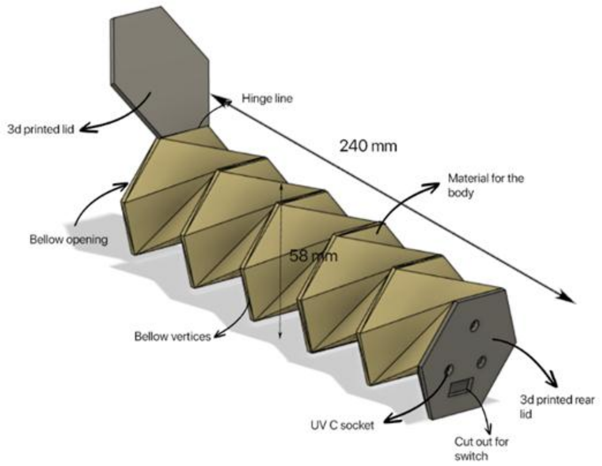

Figure 3- 3 dimensional model of the proposed device.

The design of the product has been articulated, keeping a personal point of use in mind. In remote areas, mass sanitising devices, are often controlled by local authorities with a lack of continued service, essentially producing a gap in between the recipients of the service and the health care benefit they ought to receive. This disparity has led to the pin point usage of the product i.e. to the consumers themselves. The product, being inexpensive to make due to the basic objects it uses in it construction, not only helps in the mass manufacturing process if proceeded with, but also enables, underprivileged sector to join the entire process of manufacturing to disinfection, creating a sustainable cycle [8]. Initially the prototyping time took approximately 68 hours, with a scaled up manufacturing process, the estimated time for manufacturing of 500 pieces is approximately 2 days. This numeric conclusion assumes that 5 semi-skilled labours are working in unison. Since the folding pattern has to be hand-made, the working rate consideration is 100 pieces/labour/day. The remaining plastic body shall be manufacture via injection moulding process, preferably a 4 cavity mould, to speed up production. This has been possible primarily due to the nature of design employed in this regard. Manufacturability of an origami bellow is highly dependent upon the number of fabrication folds required to make the bellow. Patterns with long, continuous folds are more manufacturable than a bellows with many short folds [3].

| Materials | Unit Cost per 10000 pcs |
|---|---|
| | |
| Paper-300 sq. cm. | $ 0.06 |
| Switch | $ 0.05 |
| UV C LED and connections | $ 0.42 |
| Plastic components | $ 0.15 |
| 3V button battery- CR2016 | $ 0.06 |
| TOTAL | $ 0.74- $ 0.95 |

Now, the next question arises with respect to the ergonomics, and how a user interacts with device. It is to be understood that the aforementioned design strategy, aims to make the entire process of using the device easy and fluid. The complexity of origami and the electrical component, have been embodied in the device for the user, in a way which masks all such complexity to bring out an easy to use product. Since disinfection is of utmost importance, the design should be able to fit in to the daily lives of the people, like something they are really comfortable to use- Once the bellow is compressed, it is shrunk to a mere disk of 12 mm height and 58 mm in diameter. This compact embodiment, fits easily inside the pocket of the consumer, making it portable not only in its expanded version but also in it compact version. Further development to the product could include a latch in the top of the front lid to attach a karabiner for hooking it up with the pant or any bag. This shall augment the ergonomic aspects of the product. However, any addition of ancillary parts to the product shall increase the cost of manufacturing and should be avoided if not necessary. The volume of the product is not rigid in nature- by altering the required parameters, the volume can either be increased or decreased depending upon the user requirement, this is primarily enables by the expanding and contracting nature of the bellow, that allows options with regard to how much volume it shall contain.

In order to use the product the user needs to pull apart both the 3D printed parts to expand the bellow- the bellow can expand without obstruction till 240 mm after which it shall start to contract. Following which he/she needs to open the front lid of the device, and insert belongings that need to be sanitised, after closing the lid, the switch connecting the UV C lamps need to be turned on and the wait for the objects to get disinfected. After which the bellow can be carefully compressed, ejecting the belongings and closing of the device, making it ready for the next use.

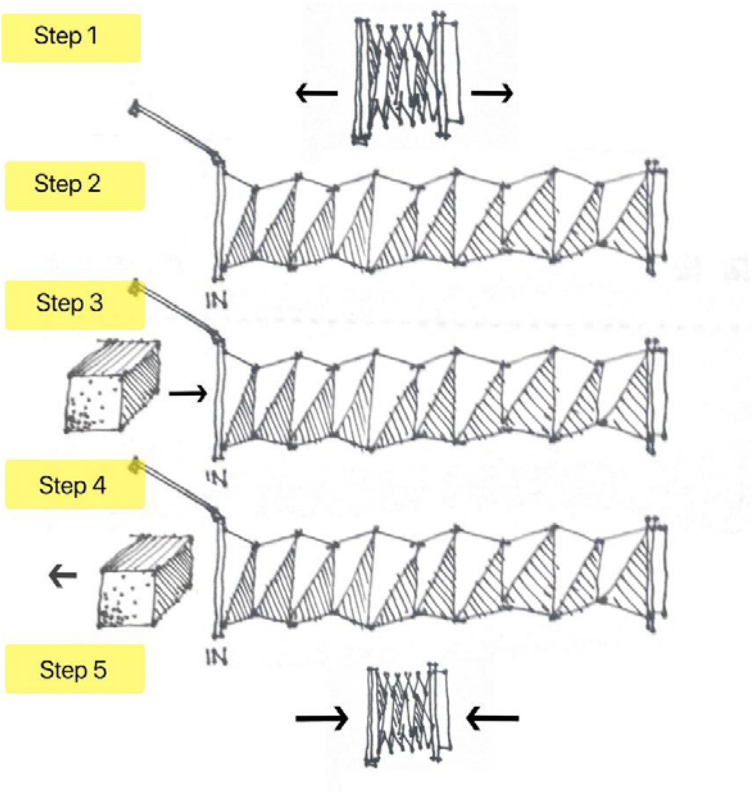

Figure 4- How the device operates.

This simplistic work mechanism of the device, is achieved by the modular product architecture it purports- where individual parts exist in modular form for them to be assembled and worked out in a convenient fashion. It is important to understand that between the conceptual and the prototyping stage, changes in types of product architecture plays a role with respect the manufacturing of the product i.e. it could have been one integrated product with built in parts for at the go manufacturing, however the expanded manufacturing line in this regard shall make the understanding of the product to the user not only more fluid but convenient.

## CONCLUSION

The various examples cited in the article uphold the process by which simple device can be designed primarily for the under privileged sector. While designing a device aligning to healthcare, in times of the SARS CoV-19 virus pandemic, there are a few hurdles that needs to be overcome, before such trying times can be tackled. As mentioned before in the article, it is of utmost importance that we devote enough attention to such factors that shall drive the development of such a product. The functional aspects and the ergonomics with regard to this product play a vital role when it comes to tackling the pandemic with the help of such devices. The parametric approach adopted while designing the product, is mainly to arrive at the best fit iteration when it comes to scale, usability and robustness- one example of such is that having many vertices in the bellow shall increase stress points in it, eventually resulting in fatigue and damage to the product, so a best fit number for the vertices was to be determined while designing it.

The product being small and compact in nature, can be easily transported as a part of mass distribution to places with dire need of it. It can act as an appropriate replacement for the expensive disinfection boxes available in the market for the design features that make it accessible to the people in several developing and underdeveloped nations- since the aspect of cost has been considered here, the raw materials being easily available can eventually augment the entire process of manufacturing and distribution.
